# Pressure distribution on a flat plate in the context of the phenomenon of the Coanda effect hysteresis

**DOI:** 10.1038/s41598-022-17031-3

**Published:** 2022-07-25

**Authors:** Aldona Skotnicka-Siepsiak

**Affiliations:** Faculty of Geoengineering, University of Warmia and Mazury in Olsztyn, Heweliusza 4, 10-724 Olsztyn, Poland

**Keywords:** Civil engineering, Fluid dynamics

## Abstract

As a result of the Coanda effect, a symmetrical free jet will flow as an asymmetrical wall jet. At the same time, at the obstacle along which the flow is observed, the wall jet generates pressure distribution. In this study, the obstacle located at the diffuser outlet is a flat plate with a variable inclination angle. The article presents results of the study on pressure distributions on a flat plate with a variable angle of inclination. In the experiment, the Reynolds number ranged from 16,192 to 42,240. A fixed geometry diffuser (Witoszyński nozzle) with a height of 0.60 m, width of 0.02 m and outlet velocity of 11.33–29.57 m/s was used. A plate with a length of 1.00 m and a variable inclination angle was installed at the diffuser outlet. What is new, however, is that the presented results of the experimental research include the influence of the Coanda effect hysteresis on the pressure distribution on the plate. The article shows how pressure distributions change on the plate depending on whether the initial angle of inclination was 0° and was increased gradually in the course of the experiment until a detachment of the jet flowing from the plate was observed, or the initial angle of inclination was close to 90° in the primal state and as the angle of the plate inclination was decreased, the jet flowing towards the plate reached the state of attachment to the plate surface. The study demonstrated that for a turbulent jet, pressure distribution on a flat plate is determined not only by the plate’s inclination angle, but also by the direction of its rotation.

## Introduction

In civil engineering, much attention is paid to the issue of a free jet. In general theoretical terms, a free jet is symmetrical. Four main zones can be distinguished in the course of the jet^1^. A short core zone, in which the axial velocity does not change, has a length of approx. four characteristic dimensions of the diffuser. Within this zone, the core of the jet can be distinguished, in which, in the axis of the jet, the initial velocity is maintained. The second one, a transition zone, is that in which the distribution of velocities in the cross-section, characteristic of free jets, arises. The length of the zone is determined by the diffuser design. The third zone is that of a fully established turbulent flow, in which there is a proportional drop in axial velocity in relation to the distance from the outlet. The zone of diffuser jet degradation is a zone of the dominant impact of the forces of internal friction, often referred to as a "die away zone", in which the axial velocity decreases rapidly, and the jet ceases to move in an orderly manner in a particular direction^2^.

There are numerous research papers dedicated to the issue of a free jet. Classical studies include, for example, papers by Corrsin^3^, Schwarc^4^, Wygnanski and Fiedler^5^ or Rajaratnam^6^. However, this issue continues to be an area of research interest. Studies that can be mentioned here include, for example, those by Lipari and Stansby^7^, who analyzed the issue of incompressible turbulent round jets issuing into a large, ideally infinite, quiescent domain. On the other hand, de la Torre et al.^8^ addressed the process of a circular free-falling jet entering an idle pool. Abdel-Rahman^9^ considered the initial and boundary conditions affecting the jet. A large number of studies use numerics for research purposes^10–12^. The issue of turbulent jet control using artificial intelligence is addressed in an article by Zhou et al.^13^.

For the formation and characteristics of a free jet, the diffuser is of key importance^14–18^. Studies by Hussein et al.^19,20^, or by Mi et al.^21^ are among the papers dedicated to the geometry of a jet generated by a round diffuser. On the other hand, examples of analyses dedicated to a rectangular nozzle include^22,23^. More atypical solutions in this regard have also been presented, e.g. for an elliptical nozzle^24,25^ or a lobed nozzle^26,27^. The diffuser shape also affects the number of symmetry axes that can be distinguished in a jet.

However, the symmetrical nature of a free jet can be very easily disturbed. For this purpose, it is sufficient to place an appropriate obstacle close enough to the jet flowing out of the diffuser, which will result in a generated wall jet. Sawyer^28^ presented two cases: where a flat plate with a variable inclination angle is attached to one of the diffuser edges at the point of jet outflow, and the outflow of a two-dimensional jet from a diffuser that is located at a distance h from the perpendicular plate. The study focused on the development of mathematical models describing the distribution of velocities and pressure and the geometry of the flow. On the other hand, Newman, in his article^29^, considers two following cases: the flow of a two-dimensional, incompressible turbulent jet around a circular cylinder and the deflection of a two-dimensional, incompressible jet under the action of a deflected flat plate. A study by Levin and Manion^30^ modified the distance of the plate location in relation to the diffuser and the inclination angle of the flat plate. The displacement of the plate in relation to the diffuser was a multiple of the gap width and amounted to 0, 2, 4, or 10, respectively. The inclination angle of the plate ranged from 0° to 55°.

A study by Lai and Lu^31^ examined the distribution of velocities and turbulence, and the location of the position of the point of jet reattachment to the plate, at different positions of its inclination. Tests were conducted for a wall jet (the plate inclination angle of α = 0°), jets at the plate inclination at an angle of α = 15°, α = 30° and α = 45°, and a free jet (the plate inclination angle of α = 90°), at Re = 10,000. The testing was conducted using a TSI IFA 100 constant-temperature anemometer. In order to determine the position of jet reattachment to the plate, measurements of pressure distribution on the plate were carried out and a visualization using threads attached to the plate was performed. Moreover, visualizations using oil film and fumigation techniques were performed. The obtained results indicated that with an increase in the plate inclination angle value, there was both a more rapid decline of the axial velocity and more rapid propagation of the jet. Consequently, due to the increase in the plate inclination angle, both the shortening of the jet core zone and an increase in the jet volume area in which entrainment occurs are observed. An extension of the research into the distribution of velocities and the turbulence of a jet adjacent to the deflectable plate to include the consideration of the distance of the deflectable plate from the diffuser can be found in an article by Nasr and Lai^32^. In the cited article, the coefficient of the plate distance from the diffuser amounted to b/D = 2.125. The testing was conducted for the plate inclination angle of α = 0° (wall jet), α = 15° and α = 30°. At the nozzle outlet, at Re = 6100. The study used a two-component Doppler laser anemometer.

Pressure distribution on a plate and pressure fluctuations play an important role in numerous technical applications, in particular in aeronautics. Meloni et al.^33^ analyzed the effect of the jet Mach number on wall pressure fluctuations caused by a compressible subsonic jet over a flat plate. Several jet Mach numbers in the range of 0.5–0.9 were analyzed. The aerodynamic characteristics of the plate effect on the jet plume were described by measuring fluid flow velocity with Pitot tubes. Two cavity-mounted pressure transducers that generate pointwise pressure signals in streamwise and spanwise directions were applied to measure wall pressure fluctuations on a flat plate. The measurements revealed that different streamwise locations induced significant changes in energy content and spectral shape. An increase in the jet Mach number influenced the amplitude, but not the shape of pressure spectra. Di Marco, Mancinelli and Camussi^34^ defined three zones of jet–plate interactions at lower Mach number (M_j_) along the jet axis. The first zone was an area where the jet was separated from the plate; the second zone denoted an area of the jet’s impact, whereas the third zone represented the transition to a quasi-equilibrium turbulent boundary layer (TBL). In addition^34^, investigated the statistical properties of wall pressure fluctuations generated by a tangential incompressible single stream jet on a rigid flat plate. Pressure measurements revealed that different stream-wise locations induced significant changes in energy content and spectral shape. The auto-spectra also differed for various axial positions, depending on the length of the flat plate. Mancinelli, Di Marco and Camussi^35^ studied the increasing size of aircraft engines and simultaneously measured velocity and wall pressure fields for different radial distances between the plate and the nozzle axis. The plate effect on velocity field statistics was measured pointwise with a hot-wire anemometer up to the fourth-order moment. The study demonstrated that the plate deflects the mean aerodynamic field over the surface and reduces turbulence intensity. Wall pressure fluctuations were also investigated by Arackal and Jothi^36^ who analyzed the effects of differently sized plates.

Preonca, Lawrence and Self^37^ examined turbulence statistics with the use of a hot-wire anemometer. They found that the mean jet flow near the surface of the plate was locally accelerated and redirected by a Coandă-type effect. The plate’s position strongly influenced the propagation of these effects downstream the plate’s trailing edge. Noise can also generate air flow. This problem was analyzed by Jorda et al.^38^ who observed the presence of tonal dynamics when an isothermal turbulent jet grazed a sharp edge. The strongest tones were generated by a coupling between Kelvin–Helmholtz wavepackets and a group of trapped, upstream-travelling acoustic modes in the potential core.

This phenomenon of deflection of the initial jet axis due to the proximity of the baffle is referred to as the Coanda effect^39^. The first written notices on observing the Coanda effect were made by Young in 1800^40^. The phenomenon was also observed by Osborne Reynolds who, in 1870, analyzed behaviors of a ball placed on the top of a vertical stream of water^41^. The Coanda effect is named after the name of the engineer Henri Marie Coanda, and was observed during his studies on the “Coanda 1910” plane, that was the first machine to use an early model of a jet engine. Presently, the phenomenon has been applied to numerous technical solutions which include respirators, dryers, blood filters and blood collecting devices, sports cars, hovercrafts, electric toothbrushes, vacuum-cleaners, cyclonic separators for air cleaning, machines for electronic chip cooling, industrial, agricultural, and automated pumps^42^.

Some results of the research into pressure distributions on a plate may also be found in numerous studies on the Coanda effect. The studies by Newman^29^, Sawyer^28^, or Lai and Lu^31^ may be provided here as examples. Those works, however, always dealt with the Coanda phenomenon and did not view the issue of pressure distribution in the aspect of hysteresis.

The phenomenon of the Coanda effect hysteresis was described for the first time by Newman^29^. It was characterized as a dependence of the state of the air jet flow (the free jet or the one attached to a flat plate is observed) on the angle of a plate inclination. In a defined range of angular α values for the inclination of a flat plate, which depend on a relation between length l of the plate and width b of a nozzle, there are two cases of air flow possible: the flow in a form of a free jet or the flow of a jet attached to the plate with an observable separation bubble.

There are few articles on the hysteresis of the Coanda effect. In the article by Allery et al.^43^, the authors described two types of own studies on occurrences of the Coanda effect hysteresis:

- the *α* value for the angle of plate inclination was changed and values of the Reynolds number for jet detachment (Re_D_) and reattachment (Re_A_); were identified
- the velocity was changed and critical values for angles of jet detachment (α_D_) and reattachment (α_A_) were identified.

The range of occurrences for the Coanda effect hysteresis reported in other sources was confirmed by experimental research, the large eddy simulation (LES), and the proper orthogonal decomposition (POD). The cited results indicate that it is enough to define values of the Re_A_ and α_A_ parameters to identify the parameters of jet attachment or reattachment for a given geometry of the system (the plate length and the nozzle width). However, to identify the parameters of jet detachment, the parameters Re_D_ and α_D_ should be defined. An analysis of the critical value of the Reynolds number in the direction of detachment makes it possible to notice, that the value increases to a small degree for the values of the plate inclination: 21° ≤ α ≤ 38°. On exceeding the value of α > 38°, it increases significantly. A similar dependence also refers to the critical value of the Reynolds number examined in the direction of the jet reattachment. There appears a significant increase of the Re_A_ value for the values of plate inclination ranging 33° ≤ α ≤ 38°.

A closer look at the range of the hysteresis occurrence area makes it possible to notice that as angular α values for plate inclination increase, there also occurs an increase of the value: ΔRe = Re_A_ − Re_D_. For the angle of plate inclination of α = 21° it is, respectively, ΔRe = 150 while for the value of α = 39° it is ΔRe = 9000. When preserving a constant velocity, as the Reynolds number increases, there also occurs an increase of the value: Δα = α_A_ – α_D_.

The study aimed to analyze the pressure distribution on a flat plate with a variable inclination angle, which is located at a flat diffuser outlet, considering the Coanda effect hysteresis. Pressure distribution on a flat plate installed at the outlet of the Witoszyński nozzle was monitored during turbulent flow. Ten air flow velocities were analyzed at plate inclination angles of 0° to 90°. The experiment was conducted in triplicate. The proposed approach is novel because it analyzes not only air velocity and the plate inclination angle, but also the direction of the plate’s rotation. For clockwise rotation, the experiment began with an inclination angle of 0° which was gradually increased. When the critical angle of jet separation α_D_ was exceeded, a wall jet was transformed into a free jet. For anticlockwise rotation, the measurements began with a plate inclination angle of 90° which was gradually decreased. When the critical angle of jet reattachment α_R_ was achieved, a free jet was transformed into a wall jet. The direction of plate rotation significantly influenced plate-jet interactions. A free jet or a wall jet was obtained for the same hydraulic parameters and plate inclination angles in the range of α_R_ > α > α_D_. Extreme differences in pressure distribution on the plate were also noted within the above range of angle values. A wall jet exerted pressure on the plate, but a free jet did not. The occurrence of a wall jet or a free jet is determined by the direction of the plate’s movement.

Under the described experimental conditions, the direction of the plate’s movement (which determines jet-plate interactions) and pressure distribution on the plate have considerable application potential in industry and engineering. Coanda effect hysteresis can be applied in solutions that rely on the Coanda effect and involve mobile elements that interact with the jet at various angles, including in aeronautics, aviation, civil and sanitary engineering.

## Experimental setup

**Figure 1:**
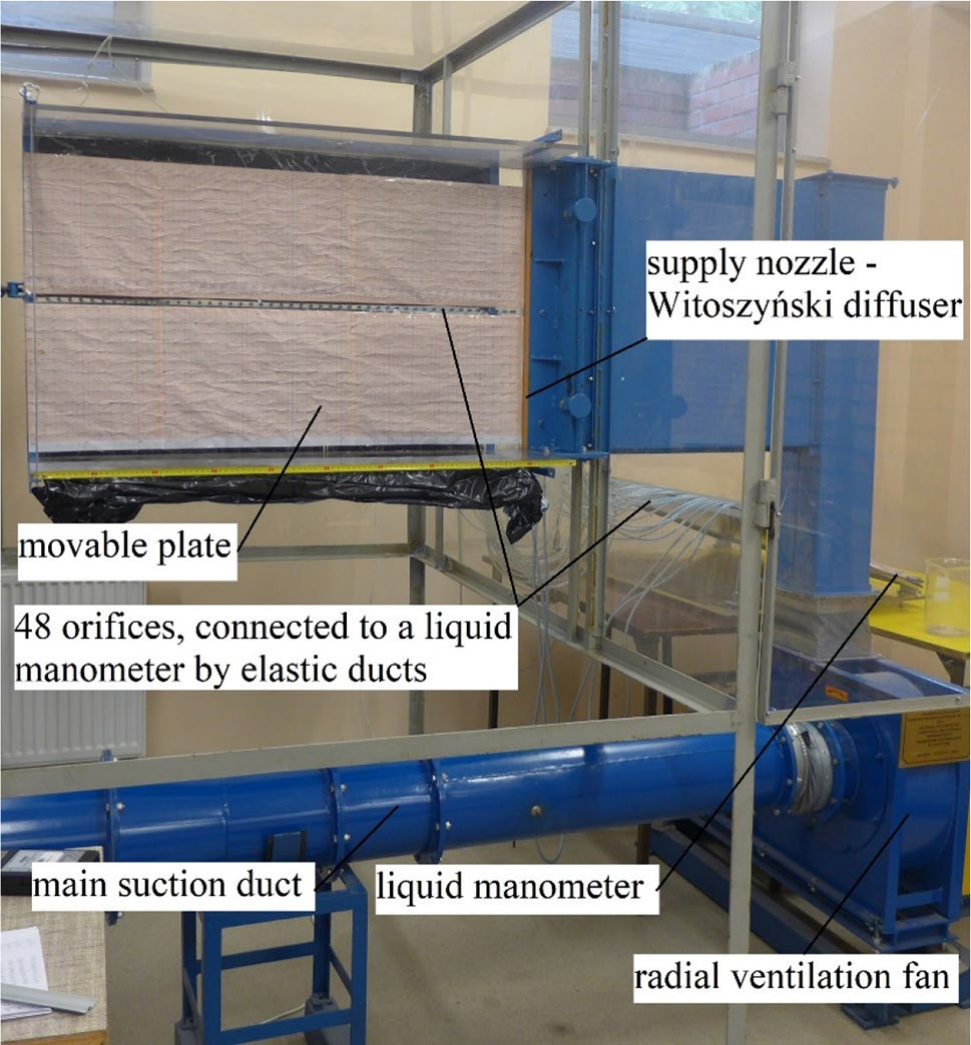
Test bench.

Own experimental studies on identifying the occurrence area of the Coanda effect hysteresis and the pressure distribution on a flat plate with a variable angle of inclination were conducted at the measuring station of the Faculty of Geoengineering at the University of Warmia and Mazury in Olsztyn (Fig. 1).

**Figure 2:**
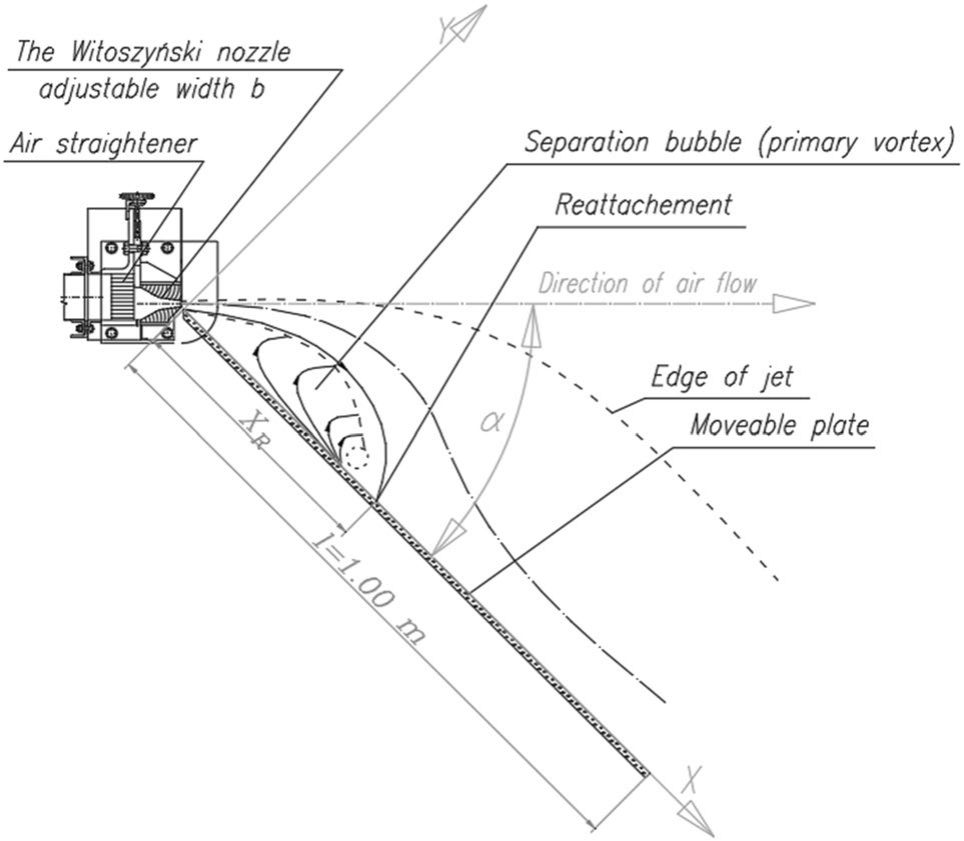
Experimental setup: Witoszyński nozzle and a plate with a variable inclination angle and variable distance x_R_ denoting the end of the recirculation zone and the point where the jet shear layer grazes over the plate surface—view from the top.

The area of the measuring station was 3.850 × 2.009 m and its height was 2.290 m. The air was delivered to the experimental system by a sucking duct with the diameter of 0.25 m and length of 5.30 m. An air intake with regulated flow and diameter of 0.40 m was mounted at the duct inlet. An orifice plate for static pressure measurements was placed in the sucking duct. The duct was connected by elastic joints to a WPO-type fan that was equipped with a three-phase fixed-gear induction motor with an EFF2-class squirrel-cage. The motor was connected to the fan by a fan belt and it was launched manually. On the pressing side of the fan, a diffuser in the shape of the Witoszyński nozzle was mounted. The height of the diffuser (h = 0.60 m) and its width (b = 0.20 m) were constant. At the nozzle outlet, a plate with a variable angle of inclination was mounted and it formed an extension of one of the diffuser edges (Fig. 2). The plate length was l = 1.00 m. The angle of the plate inclination was set manually and reading its value was possible by a scale on a rod that formed a slide for the inclined plate. The plate inclination angle was measured to the nearest ± 0.5°.

On the plate with a variable angle of inclination, there were 48 orifices, situated 20 mm away from each other, which were connected to a liquid manometer by elastic ducts. In the diffuser, there were two orifices that were also connected to the liquid manometer by elastic ducts. The liquid manometer was equipped with 51 glass tubes that were filled with a manometric liquid (alcohol) with the density of ρ = 816 kg/m^3^. The plate of the liquid manometer was inclined to the angle of β = 30° to obtain a higher accuracy of readings. The readings of the column of the manometric liquid were performed manually. An indication of the zero value was equivalent to a lack of the air flow in the research station. The accuracy of the readings was 1 mm, which corresponds to the value of 4.001 Pa.

Studies of pressure distribution on a plate were performed for ten measuring sessions with the Reynolds number ranging from 16,192 to 42,240. Identifying the *p*_*i*_ value for pressure in 48 measuring points on a plate with the length of *l* = 1.00 m was conducted using the formula:

1 $$ p_{i} = \rho gh_{i} \sin (\beta )\,\,[{\text{Pa}}] $$

on the basis of the known value of the manometric liquid density (ρ), the gravitational acceleration (g), and the experimentally determined column of the liquid in particular tubes of the liquid manometer (h_i_).

The air inlet was set manually, and air flow was set for each measurement series. The difference in pressure measured by the orifice was used to calculate flow velocity at the outlet of supply nozzle U, the volumetric flow rate and the Reynolds number.

During the studies in the direction of detachment, the plate was initially inclined to the angle α = 0°. The angle was increased gradually every 5° until the α_D_ value of the critical detachment angle was reached. The studies in the direction of the jet reattachment to the plate were started with placing the plate in the maxima angle of inclination (about 90°) when a free air flow was observed. The angle of inclination was decreased until the jet reattachment was noticed. Then, the plate inclination was discretely decreased (by multiples of 5°) until reaching the 0° value. A measurement of the pressure distribution on the plate was performed for every of the consecutive plate inclinations.

The distinction between a free jet and a wall jet was made based on the behavior of strings attached to the plate with a variable inclination angle. When the jet was attached to the plate, the strings moved in the direction of air flow. The greater the plate inclination angle, the longer the separation bubble directly behind the supply nozzle, where recirculation was confirmed by moving strings. For the wall jet, pressure on the plate was measured with a liquid manometer. The strings did not move when the free jet was not attached to the plate. In this case, pressure was not measured on the plate.

**Figure 3:**
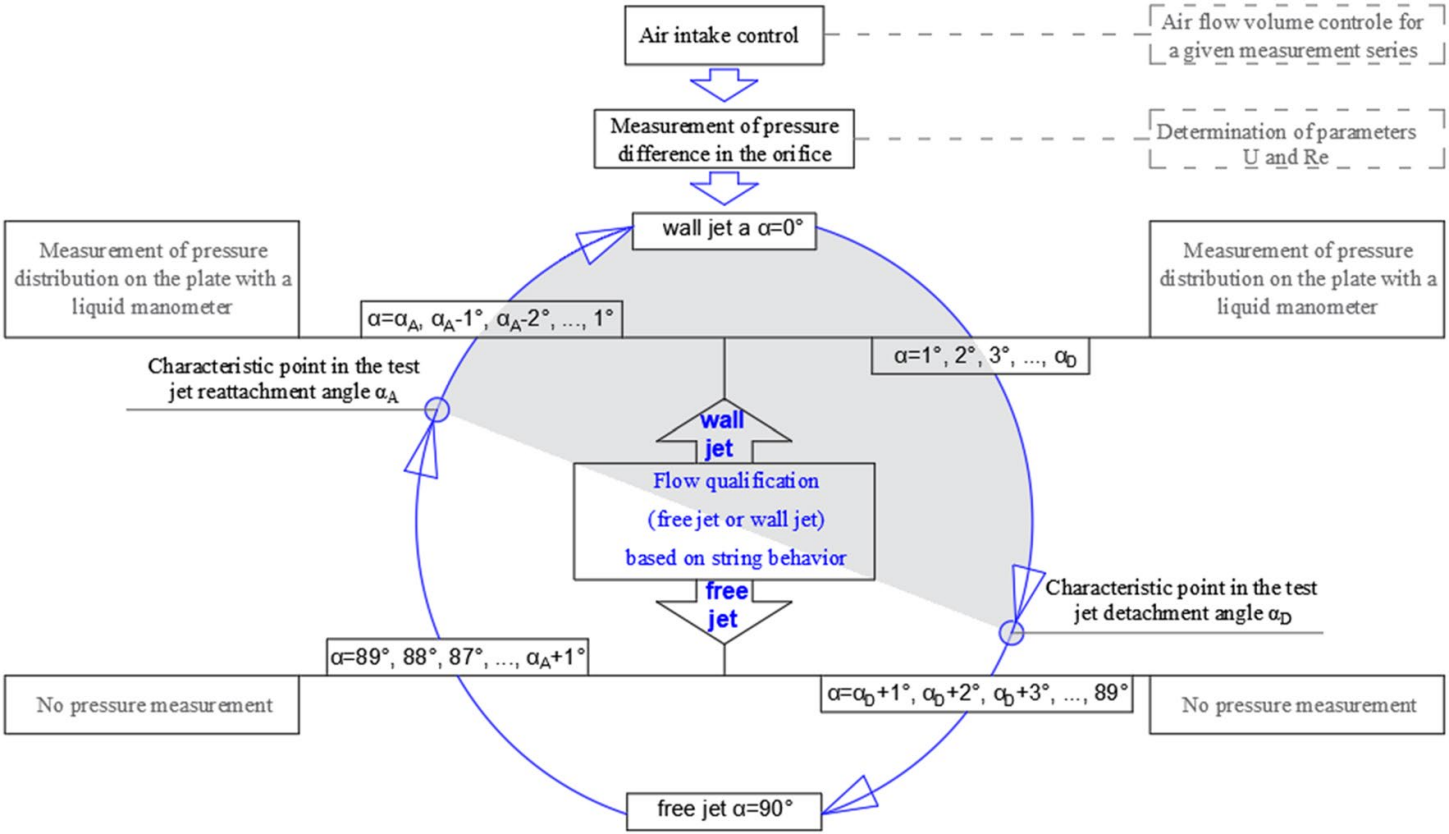
Flow chart presenting successive research tasks in the process of experimental investigations.

The experimental setup is presented in Fig. 3.

To determine air velocity, differences in air pressure in the orifice were measured with the Introl HMG 01 pressure gauge. Air velocity was measured according to standard PN-93/M-53950/01—Measurements of the mass flow rate and volumetric flow rate with Venturi tubes^44^. Based on the above standard, measurement errors were determined with the use of the below formula:

2 $$\frac{\delta q}{q}={\left[{\left(\frac{\delta C}{C}\right)}^{2}+{\left(\frac{\delta {\varepsilon }_{1}}{{\varepsilon }_{1}}\right)}^{2}+{\left(\frac{2{\beta }^{4}}{1-{\beta }^{4}}\right)}^{2}{\left(\frac{\delta D}{D}\right)}^{2}+\left(\frac{2}{1-{\beta }^{4}}\right){\left(\frac{\delta d}{d}\right)}^{2}+\frac{1}{4}{\left(\frac{\delta \Delta p}{\Delta p}\right)}^{2}+\frac{1}{4}{\left(\frac{\delta {\rho }_{1}}{{\rho }_{1}}\right)}^{2}\right]}^{1/2}$$

The measurements were conducted on the following assumptions:

- the relative uncertainty of the discharge coefficient *C* was 0.692%;
- the relative uncertainty of the dissipation rate ${\varepsilon }_{1}$ was $4\frac{\Delta p}{{p}_{1}}=0.008\%$;
- the relative uncertainty of internal tube diameter D was $\frac{\delta D}{D}=0.400\%$;
- the relative uncertainty of the throat diameter under normal conditions was $\frac{\delta d}{d}=0.070\%$;
- the ratio of the throat diameter to the main tube diameter was $\beta =\frac{d}{D}=0.692$;
- the relative uncertainty of differential pressure was $\frac{\delta \Delta p}{\Delta p}=3.858\%$;
- the relative uncertainty calculated based on the exact differential was $\frac{\delta {\rho }_{1}}{{\rho }_{1}}=0.350\%$;
- the average relative uncertainty of the volumetric flow rate was $\frac{\delta q}{q}=2.079\%$.

Air velocity at the nozzle outlet was calculated as:

3 $$U=\frac{q}{A}$$

where $U$—air velocity at the nozzle outlet [m/s], $q$—volumetric flow rate [m^3^/s], $A$—area of the nozzle outlet [m^2^].

Air velocity at the nozzle outlet ranged from 11.33 to 29.57 m/s.

The Reynolds number (Re) relative to the width of the supply nozzle and air velocity at the nozzle outlet was calculated with the use of the following formula:

4 $$\mathit{Re}=\frac{U\cdot b}{\nu }$$

where $\nu$ is kinematic viscosity [–].

## Results and discussion

**Figure 4:**
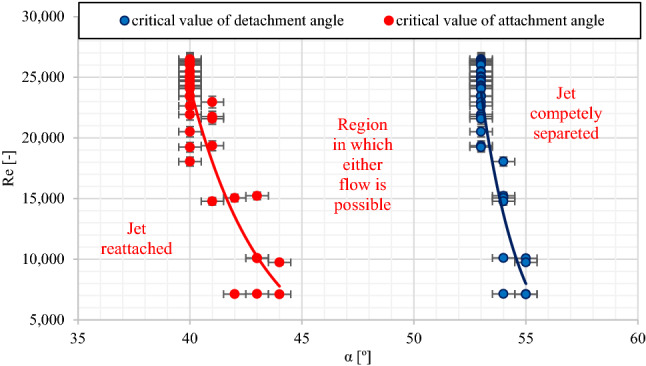
The range of occurrences for the hysteresis of the Coanda effect—critical values of the α_D_ detachment angle and the α_A_ reattachment angle.

The obtained results for the values of the critical angles of jet detachment (α_D_) and jet reattachment (α_A_) (Fig. 4) confirm the span of the area of the Coanda effect hysteresis known from the literature^45,46^. The value differences between the critical α_D_ detachment and α_A_ attachment angles are about 15°. As the Reynolds number increases, values of the critical α_D_ detachment and α_D_ attachment angles decrease.

The present results suggest that the jet behavior illustrating the Coanda effect can be explained by three factors^47^:

- the adhesion effect, namely a fluid jet’s ability to adhere to a nearby surface, and the attraction effect;
- jets flowing over convex curved surfaces or inclined plates tend to attract surrounding fluid and expand more rapidly than plane wall jets;
- a fluid jet tends to be attached to the surface when it approaches a curved surface or an inclined plate.

Newman^29^ demonstrated that jet attachment to a plate with an inclination angle α is directly caused by the forces acting upon a fluid. When a jet becomes fully detached from the plate (a free jet), the fluid from both sides of the surrounding space is entrained. Fluid that is swept up between the jet and the wall is accelerated near the plate, and pressure decreases in that region. Therefore, the pressure produced on the wall is lower than the surrounding pressure, and the lowest pressure is noted at a certain distance from the corner. As a result, the jet curves towards the wall, and when the wall is not sufficiently long, the jet will be eventually reattached. However, if spanwise flow occurs from the edge of the wall, the pressure above the plate will be roughly equal to the surrounding pressure, and the jet will not be reattached. In practice, two-dimensional jet flow is required for the above phenomenon to occur. Postman et al.^48^ found that the jet is detached from the plane when pressure on the surface of the plate (p_s_) becomes equal to external pressure (p_∞_). According to Triboix and Marchal^49^, if frictional pressure loss is neglected, the Archimedes number has to be smaller than 0.5 for the jet to become stabilized.

The pressure on the surface of the plate can be calculated as a dimensionless quantity that is dependent on the plate’s inclination angle to the axis of jet *α*, distance measured along the wall *x*, plate length *l*, width of slot *b*, and the Reynolds number^29^:

5 $$\frac{{p}_{\infty }-{p}_{s}}{{P}_{0}-{p}_{\infty }}=f\left\{\alpha ,\frac{x}{b},\frac{l}{b},Re\right\}$$

where for sufficiently high values of the Reynolds number:

6 $$Re={\left[\frac{\left({P}_{0}-{p}_{s}\right){b}^{2}}{\rho {\nu }^{2}}\right]}^{0.5}$$

When flow is entrained by the jet, the same amount of fluid is returned (by continuity) near the reattachment point within the separation bubble. Since the jet is laminar near the slot, only a small amount of fluid is entrained in that region. Therefore, the pressure on the surface of the wall (p_s_) is constant near the corner, and a minor drop in this parameter is noted when the jet becomes turbulent and when entrainment increases. The flow curvature shows that the pressure in that region is lower than the surrounding pressure p_∞_. However, wall pressure exceeds p_∞_ at the jet reattachment point and downstream, and it eventually becomes equal to p_∞_ for downstream flow.

The jet centerline can be modeled by a circular arc of radius *r* when a two-dimensional incompressible jet flow becomes reattached to a side wall that is parallel to the nozzle axis at an offset distance^50^. The pressure inside the reattached jet is equal to the minimum pressure of the separation bubble, and the relationship between the pressure difference ΔP on both sides of the jet and the centrifugal force acting on the jet can be described by the following equation:

7 $$\Delta P=\frac{J}{r}=\frac{\rho {Q}^{2}}{b{h}^{2}r}$$

where J is jet momentum, Q is the flow rate, and h is nozzle height.

The point at which a curved (under the influence of the Coanda effect) jet becomes reattached to the plate (x_R_) (Fig. 2) defines the end of the separation bubble, and it can be approximated by the following formula:

8 $${x}_{R}=2r\mathrm{sin}\;\alpha -\frac{{y}_{2}}{\mathrm{sin}\;\alpha }$$

where *r* is theoretical radius of the center line of the reattachment jet, and *y* is the distance measured perpendicular from the wall (for the coordinate system presented in Fig. 2). It is a function of the plate’s angle of inclination to the jet axis *α*, plate length *l*, slot width *b*, and the Reynolds number^29^.

The theoretical relationships describing the location of the reattachment point, the reattachment process, and the parameters of the separation bubble have been described by Epstein^51^.

**Figure 5:**
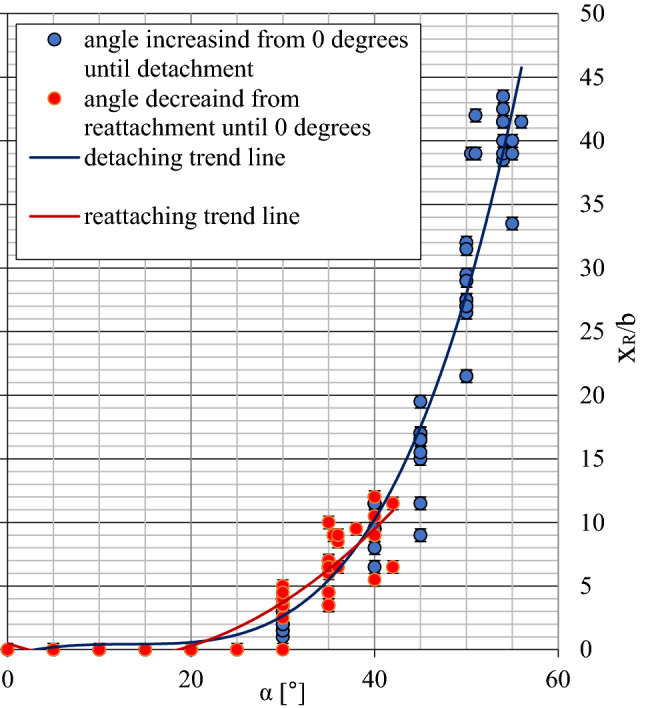
Reattachment distance x_R_ for different directions of plate movement.

The experimentally derived location of the reattachment point x_R_ relative to the nozzle (Fig. 5) indicates that the distance to point x_R_ is only somewhat greater (by around 5%) than the distance noted in the direction of detachment (α_D_) for the same values of the plate inclination angle α measured from the reattachment angle (α_A_). The width of the separation bubble increases with a rise in the plate inclination angle α. For extreme values of the jet detachment angle (α_D_), around 85% of plate length behind the nozzle falls within the region of the separation bubble.

However, theoretical assumptions rely on simplified observations. According to Newman^29^, an entrained jet behaves like a free jet in the region of the separation bubble. It is also assumed that the pressure inside the separation bubble is constant, and the jet’s center line represents the circular arc of radius r. The jet momentum is identical to the momentum of a free jet that is discharged to the atmosphere. The increase in discharge resulting from a decrease in pressure inside the bubble is neglected.

According to Görtler^52^, the values of parameter σ for calculating velocity and jet momentum should differ in the region of jet flow along the plate and inside the separation bubble, and this assumption can be considered when modeling a jet’s behavior under real-world conditions. Bubble pressure is not constant, and it increases downstream as jet velocity decreases: therefore, jet curvature cannot be constant. Mass flow is dependent on the pressure inside the separation bubble, and it increases with a drop in pressure. The pressure along the jet center streamline is not equivalent to ambient pressure because the streamline’s normal pressure gradient settles, and the center line pressure is below ambient pressure.

The described jet attachment to a plate with a variable inclination angle does not explain Coanda effect hysteresis. According to the literature^29^, the force parallel to the plate is negligible relative to jet momentum due to the skin friction of forward and backward flow, and this assumption should be taken into consideration in future research. The influence of this force is likely to increase with a decrease in the value of Re.

When examining in the direction of detachment, zero values for the pressure in almost every measuring session were recorded for the lowest values of the plate inclination of α from 0 to 15°. The only exception was the session characterized by the lowest value of the Reynolds number (Re = 16,192) for which in the initial area of the plate, directly behind the nozzle, there was an area of negative pressure of about p_i_ = − 12 Pa along a distance of approximately x/b = 5.00. Increasing the plate inclination to the angle of α = 20° resulted in appearing of a small area (x/b = 0.50) of slight negative pressure (p_i_ = − 8 Pa) for the Re = 42,240 measuring session and of about x/b = 4.55 for the Re = 16,192 measuring session. When inclining the plate to the angle of α = 25°, some clearer fluctuations appeared in the pressure values in the initial area of the plate. For the measuring session with the highest value of the Reynolds number, the positive pressure value of p_i_ =  + 8 Pa was noted which rapidly fell down to p_i_ = − 24 Pa in the distance x/b = 2.00 away from the nozzle and then increased and got normalized on the level of p_i_ = 0 Pa in the distance x/b = 4.00. As for the remaining measuring sessions, in the area of small negative pressure values, the value directly behind the nozzle fell down and stabilized on the level of p_i_ = 0 Pa in the distance x/b = 4.00 away from the nozzle. For the measuring session characterized by the lowest initial value of Re = 16,192, the constant pressure value remained on the level of p_i_ = − 12 Pa within the section of about x/b = 10.00 and then increased rapidly and stabilized on the level of p_i_ = 0 Pa.

Increasing the angle of the plate inclination to the value of α = 30° resulted in extending the area where the pressure fluctuated from the zero value. For all the measuring sessions, positive pressure values were noted directly behind the nozzle and then they decreased rapidly reaching maximum negative values characteristic for a given inclination in the distance of about x/b = 0.25 from the nozzle. In the further part of the flow, a gradual increase of the pressure value was observed and it got normalized on the level of p_i_ = 0 Pa in the distance of about 0.15 m. In the case of inclining the plate to the angle of α = 35°, the area at the beginning of the plate, which is characterized by positive pressure values, was extended to about 0.08 m. For the measuring session with the highest value of the Reynolds number, the maximum pressure value was noted in the distance of x/b = 0.20 from the nozzle. For the remaining measuring sessions, the maximum pressure values were noted directly behind the nozzle. In the further part of the flow, a rapid decrease in the pressure was visible and it reached critical values in the distance of about x/b = 6.00 from the nozzle. Further on, an increase of the pressure was noticed on the plate until it reached the value of 0 Pa in the distance of about x/b = 12.00 from the nozzle. For the measuring series with Re < 41,855, when the plate was inclined to the angle of α = 40°, maximum positive pressure values were recorded—those were the maxima for all the sessions at various plate inclinations. An extension of the area of high pressure at the initial part of the plate was visible, where a slight increase of the pressure to the maximum p_i_ values was observed. They were reached in the x/b of about from 4.00 to 6.00 behind the nozzle. Then, a rapid decrease in the pressure values was observed, until it reached the maximum recorded negative values at the given inclination angle in the x/b of about from 9.00 to 10.0 away from the nozzle. In the further part of the flow, the pressure increased to a constant value close to 0 Pa that was reached in the distance of about 0.36 m. In the case of five measuring sessions, a normalized, constant pressure value in the further area of the plate reached p_i_ from − 4 to − 8 Pa. Further increasing of the angle of the plate inclination to the values of α = 45° and α = 50° resulted in an extending the area where varied pressures occurred and in moving localizations of the points characterized by the highest and the lowest recorded pressure values away from the nozzle, as well as moving away the beginning of the area where the pressure got stabilized on the plate.

**Figure 6:**
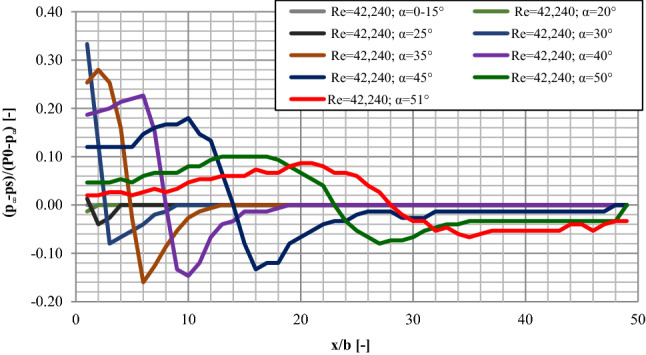
The pressure distributions on the plate—examined in the direction of detachment Re = 42,240; (where static pressure (ps); the pressure of maximal airstream (P_0_); static pressure of the surrounding fluid at rest (p∞)).

As for the plate inclination to the angle of α = 50°, the value of the pressure stabilized on the level of about p_i_ = − 20 Pa in the x/b of about 35.00 away from the nozzle. When the plate inclination was α = 45°, the pressure got stabilized on a stable level of from 0 to − 8 Pa somewhere in the middle of the plate length (about 0.50 m). A decrease in maximum and minimum pressure values as the angle of the plate inclination increased was visible at the same time. As for the plate inclination to the critical detachment angle, the value of which was dependent on the Re values for particular sessions, stable pressure values were visible in the initial area of the plate (x/b up to 4.00). For the measuring session with the lowest values of Re = 16,192, some negative pressure values were observed. For the Re = 24,284 and Re = 29,737 measuring sessions, the initial value of the pressure was 0 Pa. In the further distances from the nozzle, a gradual increase of the pressure took place until the length of x/b = 20.00 was reached. As the distance from the nozzle was increased, a decrease in the pressure was noticeable until it reached maximum negative pressure values in the distance of about 0.70 m. Some stable, negative pressure values, on the level of about p_i_ = − 35 Pa remained then until the final sections of the plate. Within the final x/b = 2.00 of the plate, there was a slight increase of the pressure to the value of about p_i_ = − 20 Pa. A sample graph for the pressure distribution on the plate with the variable angle of inclination when examined in the direction of detachment has been presented in Fig. 6.

Initially, when examining in the direction of attachment, a flow of free jet was observed at large angles of the plate inclination (about 90°), which resulted in the zero values for the column of the manometric liquid and the pressure. First readings of the manometric liquid column height value different to 0 were taken for the critical value of the jet attachment angle. In that study case, an increase of the pressure was visible and it reached its maximum value in the distance of about 0.10 m away from the nozzle. Then, a rapid decrease of the pressure occurred, until it reached the maximum recorded negative values in the distance of about 0.14 ÷ 0.20 m away from the nozzle. Then, the pressure got stabilized on the level of about p_i_ = 0 Pa. Only for the measuring session with the lowest value of Re = 16,192, the stabilized pressure remained on the level of p_i_ = − 8 Pa. At the plate inclination of 35°, which was the critical α_A_ value of the jet reattachment angle for two measuring sessions, a reduction of the area of the pressure fluctuations was visible. The pressure stabilized on the level of about p_i_ = 0 ÷ − 4 Pa in the distance of about 0.20 ÷ 0.36 m away from the nozzle.

**Figure 7:**
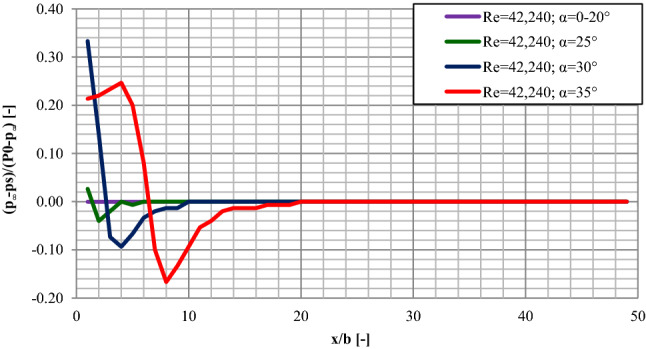
The pressure distributions on the plate—examined in the direction of attachment Re = 42,240; (where static pressure on the surface of the inclined flat plate (ps); stagnation pressure of the fluid supplying the jet (P_0_); static pressure of the surrounding fluid at rest (p∞)).

For the majority of the measuring sessions, a decrease in the pressure values occurred directly behind the nozzle, until it reached the minimum pressure value in the distance of about 0.12 m away from the nozzle. Then, a gradual increase of the pressure was observed, until it got stabilized on the level of about 0 Pa. As the angle of the plate inclination was decreased more, a further reduction of the area of pressure fluctuations was visible, as well as stabilization of the pressure values on the normalized level of about 0 Pa. At first, the recorded positive value of the pressure fell down rapidly to the maximum observed negative value. Then, it increased gradually and stabilized on the level of about 0 Pa in the distance of about 0.20 m away from the nozzle. Further decreasing the angle of the plate inclination by consecutive 5° until the value of α = 25° showed some positive pressure values in the initial area of the plate for the measuring series with the highest values of the Reynolds number (Re = 42,240 ÷ 40,075). The pressure fell down reaching the maximum negative values in the distance of about 0.04 m away from the nozzle and then increased and stabilized on the level of 0 Pa in the distance of about 0.12 m. As for the remaining measuring sessions, the zero values were recorded along the whole length of the plate. For the angles of the plate inclination ranging α = 0° ÷ 20°, the zero pressure values were observed along the whole plate. A sample graph for the pressure distribution on the plate with the variable angle of inclination when examined in the direction of attachment has been presented in Fig. 7.

**Figure 8:**
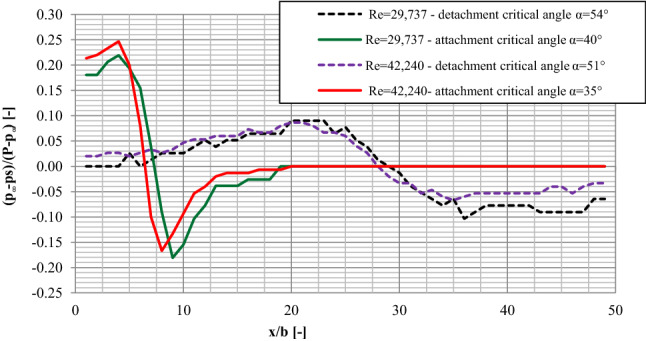
The comparison of the pressure distributions on the plate for critical attachment and detachment angles for selected measuring sessions with Re = 29,737 and Re = 42,240.

A comparison of pressure distributions on a plate for the values of critical detachment and attachment angles (Fig. 8) shows that when examining in the direction of detachment, a much longer area of pressure fluctuations on the plate was noticed and, practically, it includes its total length. When studying the critical attachment angles, the pressure was stabilized on the level of about p_i_ = 0 Pa already in the distance x/b = 20.00 away from the nozzle. Also, when examining in the direction of attachment, much higher positive and negative values were recorded for the critical angles than in the case on the same values of the Re number when placing the plate to the critical detachment angles.

When analyzing the results of the examinations in the direction of the jet reattachment, it is interesting that the maximum pressure values recorded in that study were noticed for the same values of the angle of the plate inclination for which maximum pressure values were recorded in the examinations in the direction of detachment. The minimum pressure values in the examination in the direction of reattachment were noticed when the plate was inclined to the critical α_A_ reattachment angle and they came very close to the minimum values recorded in the examinations in the direction of detachment both in respect of the angle of the plate inclination and the pressure values.

**Figure 9:**
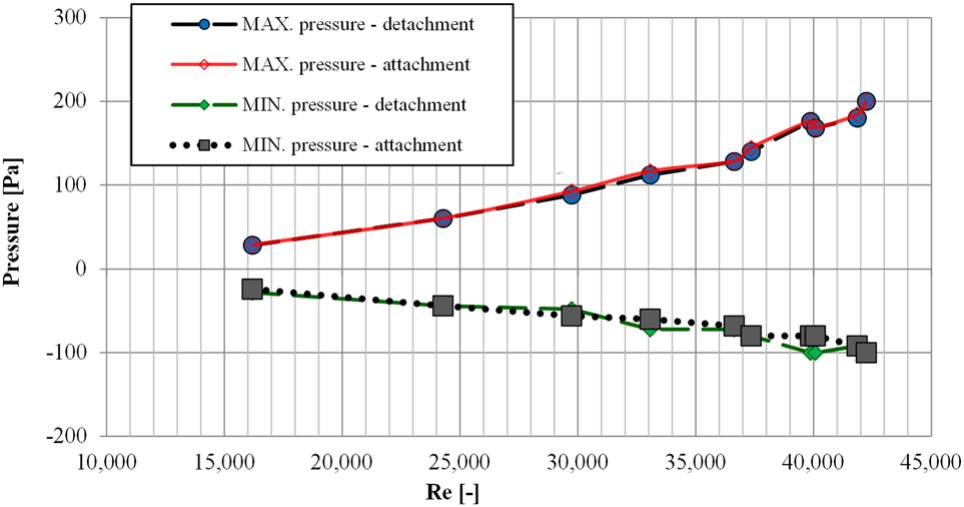
The minimum and maximum values of pressure p_i_ for the examinations in the direction of detachment and attachment depending on the value of the Reynolds number.

It can be noticed that for the discussed minimum and maximum pressure values that were recorded in the examinations in the direction of detachment or in the direction of attachment, not only the values for the angle of the plate inclination that they were recorded at in particular sessions are in accordance. Their values within particular measuring sessions are also convergent. For a study with a defined value of the Reynolds number, the maximum and minimum pressure values recorded in the examinations in the direction of on detachment are in accordance with the values recorded in the examinations in the direction of the jet reattachment (Fig. 9). The analysis of the maximum and minimum recorded p_i_ pressure values makes it possible to notice that an increase of the Reynolds numbers results in an increase of the deviation from zero for the minimum and maximum pressure values recorded in the examinations in the direction of detachment or in the direction of attachment. As for the study characterized by the highest Reynolds number (Re = 42,240), the difference between the maximum and minimum pressure value recorded for the whole study was about 300 Pa. In the case of the study with Re = 16,192, that difference was only about 52 Pa.

**Figure 10:**
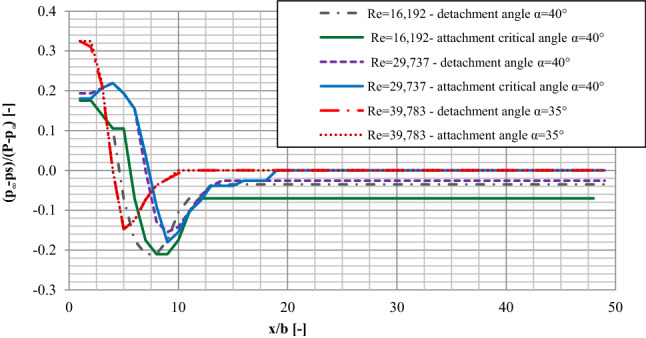
The comparison of pressure distributions on the plate at the same value of the plate inclination for the examinations in the direction of detachment and attachment.

The noticed convergence of angular range of occurrences and the maximum and minimum values of pressure for the examinations in the direction of detachment and attachment makes it interesting to compare the pressure distribution on the plate in both studies at the same value of the plate inclination (Fig. 10).

It may be noticed that, regardless of examining in the direction of detachment or attachment, if the Coanda effect was noticed on the plate, the same course of the curve for the pressure distribution on the plate was observed for the same values of the angle of the plate inclination.

Coanda effect hysteresis plays a key role in pressure distribution on a plate with an inclination angle of α_R_ > α > α_D_. When the plate inclination angle was decreased within the above range of values, the jet was detached from the plate and the pressure on the plate equaled zero. When the plate inclination angle was increased from α = 0° to α_D_, pressure initially increased and then decreased on the surface of the plate, and the differential pressure zone was elongated until the critical angle of jet separation α_D_ was reached. Pressure distributions on the plate were comparable for plate inclination angles of α < α_R_, regardless of whether the analysis focused on jet attachment or jet separation. Comparable pressure distributions were noted for the same plate inclination angles.

A comparison of pressure distributions on the plate for critical angles α_R_ and α_D_ revealed that the differential pressure zone was much longer and covered nearly the entire length of the plate when measurements were conducted from α = 0° to α_D_. When measurements were conducted from α_R_ to α = 0°, pressure was stabilized at around 0 Pa already at a distance of around 0.40 m from the supply nozzle. Positive and negative pressure was higher for critical values of α_R_ than for the same values of Re when the plate inclination angle was α_D_.

## Conclusion

Hysteresis has got a very strong influence on the range of occurrences of the Coanda effect on a flat plate. An air jet can flow as a one that is attached to the surface of a flat plate (which results in fluctuations of the pressure values the on a plat plate) or as a free one (which is of no influence on the distribution of the zero pressure values the on a plate). Depending on the initial value and direction of the change in the angle of plate inclination, three areas of angular values may be distinguished, which determine both the behavior of an air jet in the context of the Coanda effect hysteresis, as well as the pressure distribution on a flat plate:

- an area where an attachment of the jet to the plate surface is always visible—it is the area with the lowest angular α values for the plate inclination, lower than the values of the critical reattachment angle α < α_A_;
- an area where a free air flow, with no interaction with the flat plate, is always visible—the area occurs for the highest ranges of the α angle of the plate inclination, for angles higher than the value of the critical detachment angle α > α_D_;
- an area where both, a free air flow and an attachment to the plate surface, may be observed—the range of that area is about 15°. As the values of the Reynolds number decrease, the critical values for the α_D_ detachment angle and the α_A_ attachment angle increase for the flow.

The pressure distributions observed on the surface of a flat plate in the first area, where the angle of plate inclination is α < α_A_, are independent from the hysteresis of the Coanda effect. The initial value for the α angle of plate inclination nor the direction of the change of the α angle have no influence on the obtained results of pressure distribution. Regardless of examining in the direction of detachment or attachment, analogical results for the pressure distribution are obtained for the same values of the angle of plate inclination.

The phenomenon of the Coanda effect hysteresis is of key importance for the pressure distribution on a flat plate in the last of the aforementioned areas (α_A_ < α < α_D_). When examining in the direction of the air jet reattachment, the jet in that area flows regardless of the plate, on which the zero values are recorded for the pressure. However, when examining in the direction of detachment, as the α angle of plate inclination increases, a gradual decrease in the pressure on the plate surface is visible and the area of varied pressure becomes longer until a critical value for the α_D_ detachment angle is reached.

Occurrences of the Coanda effect hysteresis also result in obtaining different pressure distributions on a plate surface inclined to the critical α_D_ detachment and α_A_ attachment angles.

## Data Availability

All data generated or analyzed during this study are included in this published article.
